# Impact of Depth of Invasion in Node‐Negative Oral Tongue Cancer Treated With Surgery Alone

**DOI:** 10.1002/kjm2.70102

**Published:** 2025-09-01

**Authors:** Po‐Wen Hsiao, Yu‐Tsai Lin, Hui‐Ching Chuang, Chun‐Yuan Chao, Chih‐Yen Chien, Chao‐Hui Yang, Fu‐Min Fang, Hui Lu, Ming‐Hsien Tsai

**Affiliations:** ^1^ Department of Otolaryngology Kaohsiung Chang Gung Memorial Hospital and Chang Gung University College of Medicine Kaohsiung Taiwan; ^2^ Graduate Institute of Clinical Medical Sciences College of Medicine, Chang Gung University Taoyuan Taiwan; ^3^ School of Medicine Chang Gung University College of Medicine Taoyuan Taiwan; ^4^ School of Medicine College of Medicine, National Sun Yat‐sen University Kaohsiung Taiwan; ^5^ Doctoral Program of Clinical and Experimental Medicine National Sun Yat‐sen University Kaohsiung Taiwan; ^6^ Department of Radiation Oncology Kaohsiung Chang Gung Memorial Hospital, Chang Gung University College of Medicine Kaohsiung Taiwan

**Keywords:** depth of invasion, nomogram, oral tongue cancer, recurrence

## Abstract

Oral tongue squamous cell carcinoma (OTSCC) is an aggressive malignancy and the most common subsite of head and neck cancer among Taiwanese males. This study aimed to evaluate the prognostic significance of depth of invasion (DOI) in patients with node‐negative OTSCC treated with radical surgery alone. We retrospectively analyzed 243 patients with node‐negative OTSCC who had undergone radical surgery with adequate margins between 2005 and 2017. Each millimeter increase in DOI was significantly associated with a higher hazard of all‐cause mortality (ACM) (hazard ratio [HR], 1.07; 95% confidence interval [CI], 1.03–1.111; *p* < 0.001), cancer‐specific mortality (CSM) (HR, 1.087; 95% CI, 1.04–1.136; *p* < 0.001) and local recurrence (LR) (HR, 1.081; 95% CI, 1.021–1.145; *p* = 0.008), but not regional recurrence (RR) (HR, 1.042; 95% CI, 0.986–1.102; *p* = 0.144). In multivariate analysis, DOI remained an independent predictor of ACM, CSM, and LR. A DOI‐based nomogram demonstrated improved predictive performance, with a concordance index of 0.700 for overall survival. In conclusion, DOI represents a crucial prognostic factor for ACM, CSM, and LR in patients with node‐negative OTSCC treated with surgery alone, highlighting its potential clinical utility for early risk stratification and guidance in decision‐making regarding adjuvant therapy or intensified surveillance.

## 1 Introduction

Oral squamous cell carcinoma (OSCC) is an aggressive head and neck malignancy and the fourth most common cancer among Taiwanese males [1]. The oral tongue is the most frequently affected subsite, and oral tongue squamous cell carcinoma (OTSCC) is associated with poorer survival outcomes compared to OSCC in other subsites [2]. Surgical resection remains the standard treatment for all stages of resectable OTSCC; nevertheless, as local invasion of the primary tumor and cervical nodal metastasis are major contributors to treatment failure, postoperative adjuvant therapy is often recommended to reduce recurrence rates and improve survival in tumors with adverse pathological features [3, 4].

In the eighth edition of the American Joint Committee on Cancer (AJCC) [5], depth of invasion (DOI) was incorporated as a criterion influencing T classification in OSCC. Previous studies have demonstrated that increasing DOI is an independent predictor associated with higher rates of local and regional recurrence, as well as poorer prognosis in OTSCC [6, 7, 8, 9], the interpretation of these findings might be influenced by confounding factors such as the presence of nodal disease and the administration of adjuvant therapy. To date, few studies have examined the impact of DOI in nodal‐negative OTSCC patients undergoing radical surgery alone. Recently, Dang et al. investigated a cohort of 51 nodal‐negative OTSCC patients and found that increasing DOI was associated with higher rates of local recurrence and poorer survival [10]. This relatively homogeneous cohort provided valuable insights into the prognostic significance of DOI, though some patients received adjuvant treatment. Building on their findings, our study aims to further clarify the role of DOI in a strictly surgery‐only cohort.

The purpose of this study is to determine whether DOI is a key predictor of local and regional recurrence while investigating its impact on survival in nodal‐negative OTSCC patients treated with radical surgery alone. Additionally, we developed a nomogram incorporating DOI and other clinicopathological features to assess its utility in estimating three‐year and five‐year overall survival (OS) rates.

## 2 Materials and Methods

### 2.1 Study Design

A total of 629 previously untreated, first primary OTSCC patients underwent primary curative surgery with or without neck dissection at Kaohsiung Chang Gung Memorial Hospital, Taiwan, between January 2005 and January 2017. For patients who did not undergo neck dissection, clinical N0 classification was confirmed through preoperative imaging, ensuring the absence of enlarged lymph nodes.

**FIGURE 1:**
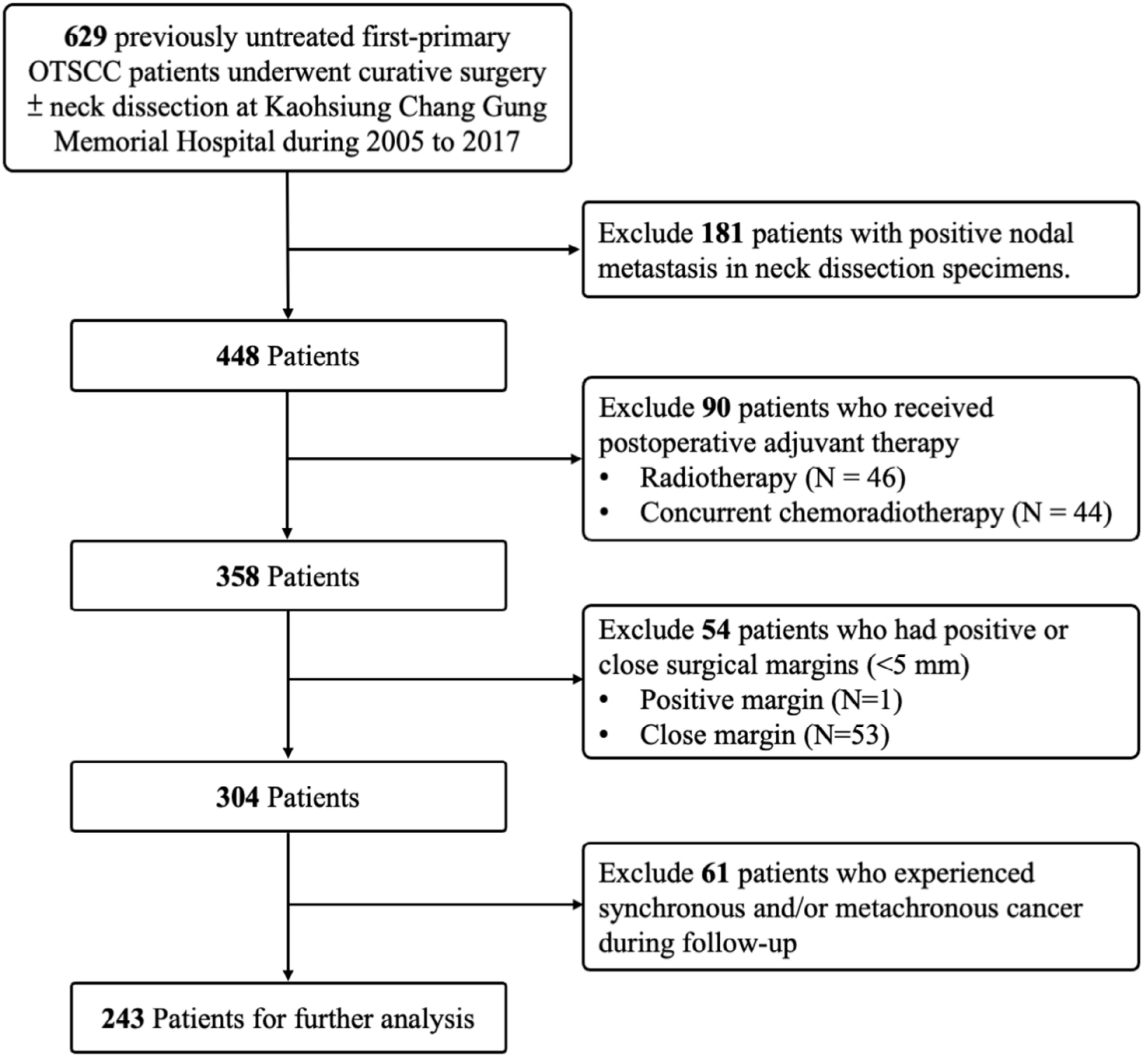
Flow diagram of patient inclusion and exclusion criteria.

To establish a homogenous patient cohort for evaluating DOI as a significant prognostic factor in oncological outcomes, we excluded patients who (a) had positive nodal metastasis in neck dissection specimens (*n* = 181), (b) received postoperative adjuvant therapy (*n* = 90), (c) had positive or close surgical margins (< 5 mm) (*n* = 54), or (d) experienced synchronous and/or metachronous cancer during follow‐up (*n* = 61). The detailed flow chart is illustrated in Figure 1. In total, 243 consecutive patients with nodal‐negative OTSCC who underwent radical surgery without postoperative adjuvant therapy were retrospectively collected and analyzed. All included patients had comprehensive clinical and pathological data available for review. This study was approved by the Medical Ethics and Human Clinical Trial Committees at Chang Gung Memorial Hospital (Ethical Application Reference number: 202500500B0).

### 2.2 Variables and Outcomes

The collected clinical and pathological variables included age, gender, lifestyle factors (alcohol consumption, betel quid chewing and smoking), tumor size, DOI of the primary tumor, histologic differentiation, perineural invasion (PNI), lymphovascular invasion (LVI), surgical margin status (all of which were assessed on the formalin‐fixed paraffin‐embedded tissue block) and the extent of neck dissection. The primary outcomes of interest were all‐cause mortality (ACM), cancer‐specific mortality (CSM), local recurrence (LR), regional recurrence (RR), and associated predictive factors. Frequencies were calculated to describe demographic and clinicopathologic characteristics, with the time frame measured from the date of radical surgery to the occurrence of each event.

### 2.3 Statistical Analysis

Continuous data were presented as medians (range), while categorical variables were expressed as numbers (frequency). The Cox proportional hazards model was used to perform both univariate and multivariate analyses for all variables, with hazard ratios (HRs) and 95% confidence intervals (CIs) calculated for each predictor. Statistical analyses were conducted using SPSS 25.0 software (SPSS/IBM Inc., Chicago, IL), with a two‐tailed *p*‐value < 0.05 considered statistically significant. Additionally, a prognostic nomogram was developed using the R software “rms” package (version 5.1–0, Vanderbilt University, Nashville, TN, USA) to predict 3‐year and 5‐year OS rates. The accuracy of the nomogram was assessed using the concordance index (C‐index) for both the conventional TNM staging system and the proposed DOI‐based model, where values of 0.5 and 1.0 indicated random and perfect predictability, respectively. Calibration plots were used to assess the agreement between predicted and actual survival outcomes.

## 3 Results

### 3.1 Patient Characteristics

The clinical characteristics of the study patients are summarized in Table 1. The median age was 53 years (range: 23–85 years), with 210 (86.4%) male and 33 (13.6%) female patients. Histologically, most tumors were moderately differentiated squamous cell carcinoma (*n* = 119, 49.0%), followed by well‐differentiated squamous cell carcinoma (*n* = 117, 48.1%) and poorly differentiated squamous cell carcinoma (*n* = 7, 2.9%). For pT classification, 131 (53.9%) tumors were classified as pT1, 67 (27.6%) as pT2, 34 (14%) as pT3, and 11 (4.5%) as pT4a. PNI and LVI were present in 12.8% and 4.1% of patients, respectively. A total of 172 (70.8%) patients underwent neck dissection, while 71 (29.2%) opted for a watchful waiting strategy for clinical nodal‐negative neck management. The median tumor size and DOI were 15 mm (range: 1–80 mm) and 4 mm (range: 1–40 mm) respectively, with the distribution of DOI being illustrated using 1‐mm intervals, as shown in Figure S1. The 5‐year overall survival rate was 87.6%, and the cancer‐specific survival rate was 93.5%, while the median follow‐up duration was 70 months.

**TABLE 1:** Patient characteristics.

Characteristics		Value	%
Median age (range), years		53 (23, 85)	
Median value of depth of invasion (range), mm		4 (1, 40)	
Median value of tumor size (range), mm		15 (1, 80)	
Median surgical margin (range), mm		7 (5, 23)	
Median follow up time (range), months		70 (1, 172.3)	
Sex	Male	210	86.4
	Female	33	13.6
Histologic grade	WDSCC	117	48.1
	MDSCC	119	49
	PDSCC	7	2.9
Perineural invasion	Absent	212	87.2
	Present	31	12.8
Lymphovascular invasion	Absent	233	95.9
	Present	10	4.1
Neck management	Neck dissection	172	70.8
	Watchful waiting	71	29.2
Recurrence	No	211	86.9
	Local recurrence only	11	4.5
	Regional relapse only	17	7
	Both locoregional failure	4	1.6

Abbreviations: MDSCC, moderately‐differentiated squamous cell carcinoma; PDSCC, poorly‐differentiated squamous cell carcinoma; WDSCC, well‐differentiated squamous cell carcinoma.

### 3.2 All‐Cause Mortality and Cancer Specific Mortality

During the follow‐up period, 37 patients (15.2%) died, including 19 (7.8%) from tongue cancer. The associations between clinicopathological characteristics and ACM and CSM are presented in Tables 2 and 3. In univariate analysis, every millimeter increase in DOI was associated with a higher hazard of ACM (HR 1.07, 95% CI: 1.03–1.111, *p* < 0.001) and CSM (HR 1.087, 95% CI: 1.04–1.136, *p* < 0.001). The presence of PNI also significantly increased the hazard of ACM and CSM (both *p* < 0.05); additionally, increased age (*p* = 0.03) and larger tumor size (*p* = 0.039) were associated with a higher hazard of ACM.

**TABLE 2:** Univariate and multivariate analysis of all‐cause mortality.

Variables		Event	Univariate analysis		Multivariate analysis	
			HR (95% CI)	*p*	aHR (95% CI)	*p*
Age, years		37	1.043 (1.014, 1.073)	0.003^a^	1.045 (1.013, 1.079)	0.005^a^
Tumor depth of invasion (mm)		37	1.07 (1.03, 1.111)	< 0.001^a^	1.076 (1.01, 1.145)	0.023^a^
Tumor size (mm)		37	1.024 (1.001, 1.047)	0.039^a^	0.997 (0.96, 1.036)	0.877
Surgical margin (mm)		37	1.041 (0.951, 1.14)	0.386	1.039 (0.937, 1.153)	0.465
Sex	Male	31	0.757 (0.315, 1.819)	0.534	0.881 (0.338, 2.291)	0.794
	Female	6	1		1	
Histologic grade				0.329		0.52
	WDSCC	17	1		1	
	MDSCC	18	1.212 (0.621, 2.362)	0.573	1.164 (0.565, 2.397)	0.681
	PDSCC	2	3.031 (0.695, 13.224)	0.14	2.581 (0.499, 13.356)	0.258
Perineural invasion	Absent	26	1	< 0.001^a^		0.066
	Present	11	3.534 (1.742, 7.17)		2.191 (0.949, 5.056)	
Lymphovascular invasion	Absent	35	1	0.587	1	0.392
	Present	2	1.485 (0.357, 6.177)		0.462 (0.079, 2.709)	
Neck management	Neck dissection	27	1	0.49		0.551
	Watchful waiting	10	1.293 (0.623, 2.686)		0.764 (0.316, 1.848)	

Abbreviations: aHR, adjusted hazard ratio; CI, confidence interval; HR, hazard ratio; MDSCC, moderately‐differentiated squamous cell carcinoma; PDSCC, poorly‐differentiated squamous cell carcinoma; WDSCC, well‐differentiated squamous cell carcinoma.

^a^ Indicate statistically significant values.

**TABLE 3:** Univariate and multivariate analysis of cancer specific mortality.

Variables		Event	Univariate analysis		Multivariate analysis	
			HR (95% CI)	*p*	aHR (95% CI)	*p*
Age, years		19	0.998 (0.957, 1.04)	0.907	0.995 (0.948, 1.044)	0.845
Tumor depth of invasion (mm)		19	1.087 (1.04, 1.136)	< 0.001^a^	1.116 (1.049, 1.188)	0.001^a^
Tumor size (mm)		19	1.027 (0.995, 1.06)	0.096	0.989 (0.942, 1.038)	0.645
Surgical margin (mm)		19	1.016 (0.885, 1.168)	0.819	1.016 (0.859, 1.202)	0.85
Sex	Male	15	0.59 (0.196, 1.778)	0.348	0.568 (0.163, 1.979)	0.374
	Female	4	1		1	
Histologic grade				0.276		0.256
	WDSCC	6	1		1	
	MDSCC	12	2.024 (0.76, 5.395)	0.158	2.282 (0.79, 6.587)	0.127
	PDSCC	1	3.506 (0.422, 29.13)	0.246	3.405 (0.342, 33.945)	0.296
Perineural invasion	Absent	14	1	0.047^a^	1	0.502
	Present	5	2.82 (1.014, 7.84)		1.492 (0.464, 4.795)	
Lymphovascular invasion	Absent	18	1	0.737	1	0.9
	Present	1	1.412 (0.188, 10.584)		0.859 (0.079, 9.332)	
Neck management	Neck dissection	14	1	0.76		0.424
	Watchful waiting	5	0.853 (0.307, 2.368)		0.597 (0.169, 2.115)	

Abbreviations: aHR, adjusted hazard ratio; CI, confidence interval; HR, hazard ratio; MDSCC, moderately‐differentiated squamous cell carcinoma; PDSCC, poorly‐differentiated squamous cell carcinoma; WDSCC, well‐differentiated squamous cell carcinoma.

^a^ Indicate statistically significant values.

After adjusting for other clinicopathological variables, DOI remained a significant independent predictor of ACM (adjusted HR [aHR] 1.076, 95% CI: 1.01–1.145, *p* = 0.023) and CSM (aHR 1.116, 95% CI: 1.049–1.188, *p* = 0.001). Older age also emerged as an independent factor associated with increased ACM in multivariate analysis (aHR 1.045, 95% CI: 1.013–1.079, *p* = 0.005).

### 3.3 Local Recurrence and Regional Recurrence

During the follow‐up period, tumor recurrence was observed in 32 patients (13.1%), including 11 with local recurrence alone, 17 with regional recurrence alone, and 4 with both locoregional recurrence, where the overall median time to LR and RR was 11.2 and 5.1 months, respectively. The associations between clinicopathological characteristics and LR are presented in Table 4. In univariate analysis, every millimeter increase in DOI was associated with a higher hazard of LR (HR 1.081, 95% CI: 1.021–1.145, *p* = 0.008). After adjusting for age, tumor size, margin status, sex, histologic grade, PNI, LVI, and neck dissection status, DOI remained a significant predictor of LR (aHR 1.094, 95% CI: 1.025–1.167, *p* = 0.007).

**TABLE 4:** Univariate and multivariate analysis of local recurrence.

Variables		Event	Univariate analysis		Multivariate analysis	
			HR (95% CI)	*p*	aHR (95% CI)	*p*
Age, years		15	0.981 (0.936, 1.028)	0.421	0.968 (0.92, 1.018)	0.205
Tumor's depth of invasion (mm)		15	1.081 (1.021, 1.145)	0.008^a^	1.094 (1.025, 1.167)	0.007^a^
Tumor size (mm)		15	1.011 (0.971, 1.053)	0.588	0.966 (0.909, 1.027)	0.267
Surgical margin (mm)		15	0.873 (0.704, 1.081)	0.213	0.86 (0.696, 1.063)	0.164
Sex	Male	13	1.011 (0.48, 2.128)	0.977	1.204 (0.249, 5.81)	0.818
	Female	2	1		1	
Histologic grade				0.452		0.339
	WDSCC	10	1		1	
	MDSCC	5	0.502 (0.171, 1.468)	0.208	0.415 (0.129, 1.339)	0.141
	PDSCC	0	0 (0, 0)	0.985	0 (0, 0)	0.983
Perineural invasion	Absent	12	1	0.318	1	0.267
	Present	3	1.905 (0.537, 6.755)		2.317 (0.525, 10.225)	
Lymphovascular invasion	Absent	14	1	0.606	1	0.971
	Present	1	1.707 (0.224, 12.989)		0.959 (0.106, 8.669)	
Neck management	Neck dissection	12	1	0.408	1	0.292
	Watchful waiting	3	0.586 (0.165, 2.078)		2.18 (0.512, 9.281)	

Abbreviations: aHR, adjusted hazard ratio; CI, confidence interval; HR, hazard ratio; MDSCC, moderately‐differentiated squamous cell carcinoma; PDSCC, poorly‐differentiated squamous cell carcinoma; WDSCC, well‐differentiated squamous cell carcinoma.

^a^ Indicate statistically significant values.

The associations between clinicopathological characteristics and RR are presented in Table 5. In multivariate analysis, neck management strategy was the only independent factor associated with RR, while DOI did not show a significantly increased hazard of RR. Given that different neck management strategies could confound the assessment of regional failure, we conducted a subgroup analysis of DOI stratified by neck management. The median time to regional recurrence was 5.3 months (range: 2–27.8) in the neck dissection group and 5.1 months (range: 2.3–18.9) in the watchful waiting group. In univariate analysis, every millimeter increase in DOI was associated with a higher hazard of RR in the watchful waiting group (HR 1.355, 95% CI: 1.101–1.668, *p* = 0.004), DOI did not significantly increase the hazard of RR in the neck dissection group (HR 1.056, 95% CI: 0.989–1.126, *p* = 0.102) (Table 6).

**TABLE 5:** Univariate and multivariate analysis of regional recurrence.

Variables		Event	Univariate analysis		Multivariate analysis	
			HR (95% CI)	*p*	aHR (95% CI)	*p*
Age, years		21	1.01 (0.973, 1.049)	0.598	1.008 (0.967, 1.051)	0.706
Tumor's depth of invasion (mm)		21	1.042 (0.986, 1.102)	0.144	1.032 (0.912, 1.168)	0.62
Tumor size (mm)		21	1.02 (0.989, 1.051)	0.218	1.023 (0.966, 1.084)	0.437
Surgical margin (mm)		21	1.063 (0.944, 1.197)	0.315	1.132 (0.978, 1.31)	0.098
Sex	Male	16	0.702 (0.425, 1.159)	0.167	0.59 (0.191, 1.822)	0.359
	Female	5	1		1	
Histologic grade				0.049^a^		0.054
	WDSCC	6	1		1	
	MDSCC	13	2.159 (0.821, 5.681)	0.119	2.564 (0.934, 7.042)	0.068
	PDSCC	2	6.935 (1.398, 34.397)	0.018	7.824 (1.207, 50.721)	0.031
Perineural invasion	Absent	15	1	0.025^a^	1	0.28
	Present	6	2.949 (1.144, 7.602)		1.849 (0.607, 5.633)	
Lymphovascular invasion	Absent	20	1	0.896	1	0.449
	Present	1	1.144 (0.153, 8.522)		0.41 (0.041, 4.127)	
Neck management	Neck dissection	12	1	0.147	1	0.008^a^
	Watchful waiting	9	0.528 (0.222, 1.253)		0.215 (0.068, 0.675)	

Abbreviations: aHR, adjusted hazard ratio; CI, confidence interval; HR, hazard ratio; MDSCC, moderately‐differentiated squamous cell carcinoma; PDSCC, poorly‐differentiated squamous cell carcinoma; WDSCC, well‐differentiated squamous cell carcinoma.

^a^ Indicate statistically significant values.

**TABLE 6:** Subgroup analysis of depth of invasion in regional recurrence according to different neck management.

Variable	Neck management	Event	Univariate analysis	
			HR (95% CI)	*p*
Tumor depth of invasion (mm)	Watchful waiting	9	1.355 (1.101, 1.668)	0.004^a^
	Neck dissection	12	1.056 (0.989, 1.126)	0.102

Abbreviations: CI, confidence interval; HR, hazard ratio.

^a^ Indicate statistically significant values.

### 3.4 Predictive Nomogram of Overall Survival

**FIGURE 2:**
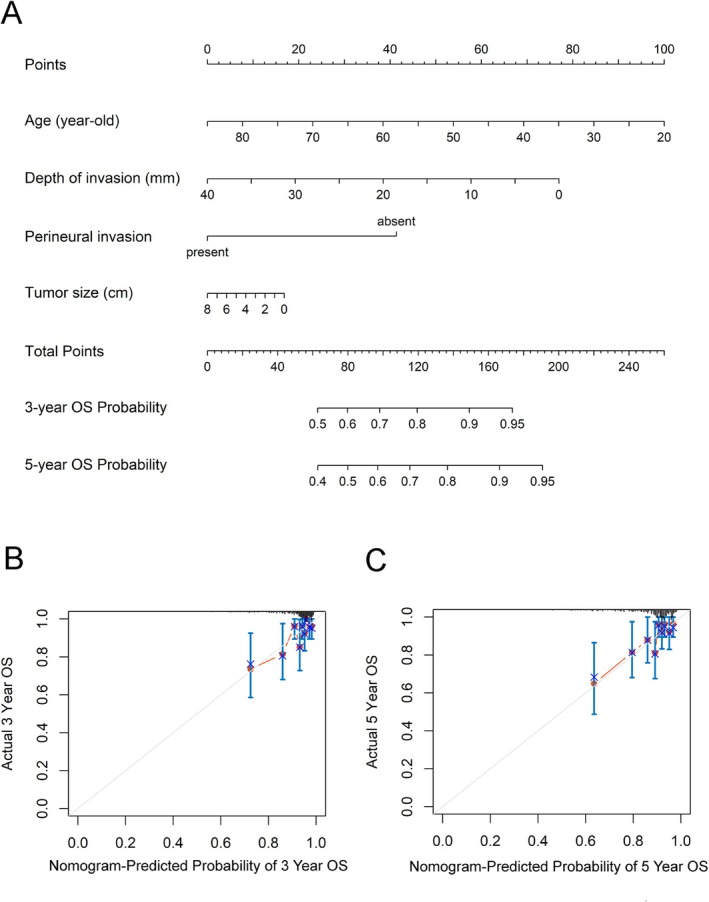
DOI‐based nomogram for OS prediction. (A) Nomogram. Calibration plots were generated for (B) 3‐year and (C) 5‐year overall survival rates.

To further validate the prognostic value of DOI, we developed a multivariate nomogram integrating significant prognostic factors identified in the univariate analysis of ACM to predict 3‐year and 5‐year OS. As shown in Figure 2A, age was the most influential prognostic factor, followed by DOI, PNI, and tumor size. Each factor contributes a specific number of points, and the total score for an individual patient is calculated by summing these points; then the corresponding 3‐year and 5‐year OS probabilities are determined using the nomogram's survival probability scale. Calibration curves demonstrated that the predicted 3‐year and 5‐year OS closely aligned with actual outcomes (Figure 2B,C). The nomogram exhibited strong predictive performance, with a C‐index of 0.700 (95% CI: 0.609–0.793), outperforming the traditional TNM staging system, which had a C‐index of 0.618 (95% CI: 0.524–0.712).

## 4 Discussion

To the best of our knowledge, this is the first study to evaluate the clinical significance of DOI in patients with nodal‐negative OTSCC who had undergone radical surgery without adjuvant therapy. Our findings demonstrated that every millimeter increase in DOI was associated with a 7% increase in the hazard of ACM (HR 1.07), an 8.7% increase in the hazard of CSM (HR 1.087), and an 8.1% increase in the hazard of LR (HR 1.081) in this patient population. In multivariate analysis, after adjusting for other variables, DOI remained a statistically significant predictor of ACM (aHR 1.076), CSM (aHR 1.116) and LR (aHR 1.094); furthermore, every millimeter increase in DOI was associated with a 35.5% increase in the hazard of regional recurrence (HR 1.355) in OTSCC patients with cN0 disease who underwent a watchful waiting strategy for neck management.

Numerous studies have demonstrated that increased DOI is associated with a higher risk of locoregional recurrence [6, 8, 9, 10, 11, 12, 13, 14]. This association might be explained by the tendency of tumors with greater DOI to exhibit adverse histopathological characteristics such as PNI and LVI, which are known to contribute to disease progression and recurrence [8, 15, 16]. In our study, increasing DOI was also found to have a statistically significant positive correlation to the presence of PNI (*r* = 0.290, *p* < 0.001) and LVI (*r* = 0.145, *p* = 0.024) based on Pearson correlation analysis. Importantly, DOI remained statistically significant in multivariate analysis after adjusting for PNI and LVI, suggesting that other intrinsic factors could contribute to recurrence. For instance, tumor budding and the worst pattern of invasion have been consistently associated with increased DOI and have also been linked to a higher rate of tumor recurrence in OSCC [17, 18, 19, 20]. Another critical factor to consider is the development of hypoxic regions as tumor invasion deepens due to limited oxygen diffusion. Hypoxia promotes the stabilization of hypoxia‐inducible factor 1‐alpha (HIF‐1α), which transcriptionally activates epithelial‐mesenchymal transition (EMT) regulators. This process enhances tumor invasion and facilitates metastasis [21, 22].

A notable finding from our cohort is that although DOI was not identified as a risk factor for RR in the overall analysis, it became a significant factor in the subgroup of patients who did not undergo neck dissection. In other words, for patients with greater DOI, neck dissection appeared to mitigate its negative impact on RR. A study with a similar cohort by Kozak et al. examined early‐stage OSCC patients who underwent surgical resection with or without neck dissection, excluding those with positive or close margins, LVI, PNI, or positive lymph nodes. Their findings similarly demonstrated that DOI (≥ 4 mm vs. < 4 mm) was not correlated with RR in the overall cohort; however, among patients who did not undergo neck dissection, the proportion of patients who developed RR was higher in those with DOI ≥ 4 mm [23]. In our cohort, focusing on patients with cN0 disease who underwent a watchful waiting strategy for neck management (*N* = 71), we found that when evaluating regional recurrence based on cumulative DOI, a marked increase in recurrence rate was observed at a cutoff of 3 mm, where the event rate rose from 0% to 6.2% (Figure S2). This sharp increase suggests that patients with DOI exceeding 3 mm might be at significantly higher risk of regional recurrence, warranting closer surveillance or consideration of additional therapeutic strategies, including staged neck dissection following primary tumor ablation. Our findings align with a nationwide study of 3886 cT1N0M0 oral cancer patients that concluded DOI > 2.5 mm should be an indication for elective neck dissection to improve survival outcomes [24].

Although the prognostic role of DOI in oral cancer survival is well established, most studies have categorized DOI into two groups using various cutoff values; in contrast, our study analyzed DOI as a continuous variable and demonstrated that every millimeter increase was associated with an elevated risk of ACM, CSM, and LR in nodal‐negative OTSCC patients who underwent radical surgery. Only a few studies have evaluated DOI as a continuous variable. Toom et al. investigated OSCC patients staged by sentinel lymph node biopsy and reported an ROC AUC of 0.65 with an optimal DOI cutoff of 3.4 mm, noting that 15% of patients with DOI ≤ 3.4 mm developed regional metastases [25]. Edri et al. found that DOI, when treated as a continuous variable, was a stronger predictor of mortality, locoregional recurrence, and occult lymph node metastasis compared to categorical analysis [26]. Similarly, Shinn et al. demonstrated a progressive increase in regional recurrence risk with DOI, suggesting that no single threshold reliably predicts nodal metastasis [13]. Collectively, these findings emphasize the importance of analyzing DOI as a continuous prognostic factor in OSCC rather than relying on predefined cutoff points.

We also found that old age was associated with an increased risk of ACM, but this trend was not observed for CSM. This finding suggests that more elderly patients passed away due to comorbid conditions rather than cancer itself. Given the significant mortality in onco‐geriatric patients, recognizing the impact of age on post‐surgical outcomes might help both patients and clinicians make more informed treatment decisions, particularly for older individuals. In this study, we developed a nomogram incorporating significant clinicopathologic predictors of OS to improve prognosis prediction in nodal‐negative OTSCC patients after radical surgery. Nomograms are widely used in oncology, including oral cancer, as reliable predictive tools that aid in patient stratification and personalized treatment planning [27, 28, 29, 30, 31, 32, 33]. However, to date, no study has constructed a DOI‐based nomogram specifically for predicting survival in nodal‐negative OTSCC. We assessed its discriminative performance using the C‐index and calibration plots. The results demonstrated that the proposed model has dependable predictive accuracy and discriminative ability.

This study has several limitations. Firstly, it is a retrospective single‐institution study in which all surgeries were performed by different head and neck surgeons; although in an attempt to minimize potential bias, we carefully selected a homogeneous patient cohort and made significant efforts to ensure that DOI was measured in a standardized manner. However, the low incidence of adverse features such as PNI and LVI, the predominance of early‐stage disease, and the overall low rate of local recurrence limit the strength of the conclusions that can be drawn. Secondly, our findings were not validated using an independent dataset. External validation with an independent patient cohort would enhance the robustness of our results and further support the role of DOI in predicting ACM, CSM, and recurrence in nodal‐negative OTSCC patients. Future studies, particularly well‐designed prospective multi‐institutional studies or analyses using national cancer databases, would help minimize these limitations and provide stronger evidence.

In conclusion, our study demonstrates that DOI is a significant predictor of ACM, CSM, and LR in nodal‐negative OTSCC patients who underwent radical surgery alone while also being associated with RR in patients managed with a watchful waiting strategy; although neck dissection appeared to reduce this risk. Furthermore, we developed a DOI‐based nomogram to estimate OS, integrating key clinicopathologic predictors. The model exhibited satisfactory predictive accuracy and could aid clinicians in patient stratification and treatment planning for nodal‐negative OTSCC.

## Conflicts of Interest

The authors declare no conflicts of interest.

## Supporting information


**Figure S1:** Distribution of tumor's depth of invasion in our cohort.
**Figure S2:**. Regional recurrence rate based on cumulative depth of invasion in subgroup patients with cN0 disease who underwent a watchful waiting strategy for neck management.

## Data Availability

The data that support the findings of this study are available from the corresponding author upon reasonable request.

## References

[1] J. R. Jhuang , S. Y. Su , C. J. Chiang , et al., “Forecast of Peak Attainment and Imminent Decline After 2017 of Oral Cancer Incidence in Men in Taiwan,” Scientific Reports 12, no. 1 (2022): 5726. 35388051 10.1038/s41598-022-09736-2 PMC8987068

[2] K. Rusthoven , A. Ballonoff , D. Raben , and C. Chen , “Poor Prognosis in Patients With Stage I and II Oral Tongue Squamous Cell Carcinoma,” Cancer 112, no. 2 (2008): 345–351. 18041071 10.1002/cncr.23183

[3] J. Bernier , C. Domenge , M. Ozsahin , et al., “Postoperative Irradiation With or Without Concomitant Chemotherapy for Locally Advanced Head and Neck Cancer,” New England Journal of Medicine 350, no. 19 (2004): 1945–1952. 15128894 10.1056/NEJMoa032641

[4] J. S. Cooper , T. F. Pajak , A. A. Forastiere , et al., “Postoperative Concurrent Radiotherapy and Chemotherapy for High‐Risk Squamous‐Cell Carcinoma of the Head and Neck,” New England Journal of Medicine 350, no. 19 (2004): 1937–1944. 15128893 10.1056/NEJMoa032646

[5] M. B. Amin , F. L. Greene , S. B. Edge , et al., “The Eighth Edition AJCC Cancer Staging Manual: Continuing to Build a Bridge From a Population‐Based to a More “Personalized” Approach to Cancer Staging,” CA: A Cancer Journal for Clinicians 67, no. 2 (2017): 93–99. 28094848 10.3322/caac.21388

[6] A. Almangush , I. O. Bello , R. D. Coletta , et al., “For Early‐Stage Oral Tongue Cancer, Depth of Invasion and Worst Pattern of Invasion Are the Strongest Pathological Predictors for Locoregional Recurrence and Mortality,” Virchows Archiv 467, no. 1 (2015): 39–46. 25838076 10.1007/s00428-015-1758-z

[7] A. Almangush , I. O. Bello , H. Keski‐Säntti , et al., “Depth of Invasion, Tumor Budding, and Worst Pattern of Invasion: Prognostic Indicators in Early‐Stage Oral Tongue Cancer,” Head and Neck 36, no. 6 (2014): 811–818. 23696499 10.1002/hed.23380 PMC4229066

[8] M. Newman , P. T. Dziegielewski , N. T. A. Nguyen , et al., “Relationship of Depth of Invasion to Survival Outcomes and Patterns of Recurrence for T3 Oral Tongue Squamous Cell Carcinoma,” Oral Oncology 116 (2021): 105195. 33618103 10.1016/j.oraloncology.2021.105195

[9] M. H. Tsai , H. S. Huang , H. C. Chuang , et al., “Patients of Stage I Oral Cancer With Pathologically Lowrisk Feature Managed by Primary Tumor Resection Alone: Impact of Depth of Invasion and a Nomogram Analysis,” Laryngoscope Investigative Otolaryngology 7, no. 4 (2022): 1025–1032. 36000051 10.1002/lio2.872 PMC9392408

[10] R. R. Dang , J. Kim , M. M. Qureshi , et al., “Impact of Depth of Invasion on Local Recurrence in R0 Resected Node‐Negative Oral Tongue Squamous Cell Carcinoma,” Head & Neck 45, no. 3 (2023): 561–566. 36513522 10.1002/hed.27264

[11] M. Faisal , M. Abu Bakar , A. Sarwar , et al., “Depth of Invasion (DOI) as a Predictor of Cervical Nodal Metastasis and Local Recurrence in Early Stage Squamous Cell Carcinoma of Oral Tongue (ESSCOT),” PLoS One 13, no. 8 (2018): e0202632. 30133515 10.1371/journal.pone.0202632 PMC6105019

[12] S. V. Kane , M. Gupta , A. C. Kakade , and D. C. A , “Depth of Invasion Is the Most Significant Histological Predictor of Subclinical Cervical Lymph Node Metastasis in Early Squamous Carcinomas of the Oral Cavity,” European Journal of Surgical Oncology 32, no. 7 (2006): 795–803. 16777368 10.1016/j.ejso.2006.05.004

[13] J. R. Shinn , C. B. Wood , J. M. Colazo , F. E. Harrell, Jr. , S. L. Rohde , and K. Mannion , “Cumulative Incidence of Neck Recurrence With Increasing Depth of Invasion,” Oral Oncology 87 (2018): 36–42. 30527241 10.1016/j.oraloncology.2018.10.015

[14] C. G. F. van Lanschot , Y. P. Klazen , M. A. J. de Ridder , et al., “Depth of Invasion in Early Stage Oral Cavity Squamous Cell Carcinoma: The Optimal Cut‐Off Value for Elective Neck Dissection,” Oral Oncology 111 (2020): 104940. 32769035 10.1016/j.oraloncology.2020.104940

[15] J. Li , S. Liu , Z. Li , X. Han , and L. Que , “Prognostic Value of Perineural Invasion in Oral Tongue Squamous Cell Carcinoma: A Systematic Review and Meta‐Analysis,” Frontiers in Oncology 11 (2021): 683825. 34322385 10.3389/fonc.2021.683825 PMC8311439

[16] M. M. Masood , D. R. Farquhar , J. P. Vanleer , S. N. Patel , and T. G. Hackman , “Depth of Invasion on Pathological Outcomes in Clinical Low‐Stage Oral Tongue Cancer Patients,” Oral Diseases 24, no. 7 (2018): 1198–1203. 29750853 10.1111/odi.12887

[17] L. Togni , V. C. A. Caponio , N. Zerman , et al., “The Emerging Impact of Tumor Budding in Oral Squamous Cell Carcinoma: Main Issues and Clinical Relevance of a New Prognostic Marker,” Cancers 14, no. 15 (2022): 3571. 35892830 10.3390/cancers14153571 PMC9332070

[18] K. Yadav , T. Singh , K. Varma , M. Bhargava , and V. Misra , “Evaluation of Tumor Budding and Its Correlation With Histomorphological Prognostic Markers in Oral Squamous Cell Carcinoma and Its Association With the Epithelial‐Mesenchymal Transition Process,” Indian Journal of Pathology and Microbiology 66, no. 1 (2023): 3–8. 36656202 10.4103/ijpm.ijpm_190_22

[19] P. Hurník , J. Režnarová , Z. Chyra , et al., “Enhancing Oral Squamous Cell Carcinoma Prediction: The Prognostic Power of the Worst Pattern of Invasion and the Limited Impact of Molecular Resection Margins,” Frontiers in Oncology 13 (2023): 1287650. 38188288 10.3389/fonc.2023.1287650 PMC10766711

[20] N. Sinha , M. H. Rigby , M. L. McNeil , et al., “The Histologic Risk Model Is a Useful and Inexpensive Tool to Assess Risk of Recurrence and Death in Stage I or II Squamous Cell Carcinoma of Tongue and Floor of Mouth,” Modern Pathology 31, no. 5 (2018): 772–779. 29393297 10.1038/modpathol.2017.183

[21] Y. Li , B. Li , K. Yang , et al., “PER3 Suppresses Tumor Metastasis of Oral Squamous Cell Carcinoma by Promoting HIF‐1α Degradation,” Translational Oncology 52 (2025): 102258. 39733745 10.1016/j.tranon.2024.102258 PMC11743850

[22] G. B. Morand , M. A. Broglie , P. Schumann , M. W. Huellner , and N. J. Rupp , “Histometabolic Tumor Imaging of Hypoxia in Oral Cancer: Clinicopathological Correlation for Prediction of an Aggressive Phenotype,” Frontiers in Oncology 10 (2020): 1670. 32984043 10.3389/fonc.2020.01670 PMC7481376

[23] M. M. Kozak , J. Shah , M. Chen , et al., “Depth of Invasion Alone as a Prognostic Factor in Low‐Risk Early‐Stage Oral Cavity Carcinoma,” Laryngoscope 129, no. 9 (2019): 2082–2086. 30604435 10.1002/lary.27753

[24] C. Y. Chien , C. P. Wang , L. Y. Lee , et al., “Indications for Elective Neck Dissection in cT1N0M0 Oral Cavity Cancer According to the AJCC Eight Edition: A Nationwide Study,” Oral Oncology 140 (2023): 106366. 36965411 10.1016/j.oraloncology.2023.106366

[25] I. J. den Toom , L. M. Janssen , R. J. J. van Es , et al., “Depth of Invasion in Patients With Early Stage Oral Cancer Staged by Sentinel Node Biopsy,” Head & Neck 41, no. 7 (2019): 2100–2106. 30688384 10.1002/hed.25665 PMC6618049

[26] N. Edri , D. Dudkiewicz , D. Yaniv , et al., “Evaluating Depth of Invasion as a Continuous Prognostic Factor in Oral Squamous Cell Carcinoma,” Head & Neck 47, no. 3 (2025): 856–866. 39474634 10.1002/hed.27979 PMC11816571

[27] V. P. Balachandran , M. Gonen , J. J. Smith , and R. P. DeMatteo , “Nomograms in Oncology: More Than Meets the Eye,” Lancet Oncology 16, no. 4 (2015): e173–e180. 25846097 10.1016/S1470-2045(14)71116-7 PMC4465353

[28] L. Chen , J. Qian , L. Lin , et al., “Prognostic Value of Preoperative Lymphocyte‐To‐Monocyte Ratio in Oral Cancer Patients and Establishment of a Dynamic Nomogram,” Oral Diseases 27, no. 5 (2021): 1127–1136. 32881142 10.1111/odi.13629

[29] H. Fei and X. Chen , “A Novel Autophagy‐Related Prognostic Risk Model and a Nomogram for Survival Prediction of Oral Cancer Patients,” BioMed Research International 2022 (2022): 2067540. 35036428 10.1155/2022/2067540 PMC8758260

[30] S. Gupta , J. Waller , J. Brown , Y. Elam , J. V. Rawson , and D. Pucar , “Nomogram Identifies Age as the Most Important Predictor of Overall Survival in Oral Cavity Squamous Cell Cancer After Primary Surgery,” Indian Journal of Otolaryngology and Head & Neck Surgery 72, no. 2 (2020): 160–168. 32551272 10.1007/s12070-019-01726-7 PMC7276464

[31] Y. Liu , Y. Ma , G. Shayan , et al., “Improved Cancer‐Specific Risk Stratification by the Lymph Node Ratio‐Based Nomogram: A Potential Role in Guiding Postoperative Management Decisions for Oral Cavity Carcinoma,” JCO Precision Oncology 7 (2023): e2200365. 36603173 10.1200/PO.22.00365

[32] N. Sun , L. Huang , H. G. Xiong , et al., “Nomogram vs. Depth of Invasion for Predicting Occult Lymph Node Metastasis in cT1‐2N0 Buccal Squamous Cell Carcinoma,” Oral Oncology 162 (2025): 107206. 39874722 10.1016/j.oraloncology.2025.107206

[33] W. Wang , Q. Zhang , P. Thomson , D. Sharma , P. Ramamurthy , and S. W. Choi , “Predicting Oral Cancer Survival‐Development and Validation of an Asia‐Pacific Nomogram,” Journal of Oral Pathology & Medicine 52, no. 7 (2023): 628–636. 37247328 10.1111/jop.13454

