# Supplementary material for: Impact of Depth of Invasion in Node‐Negative Oral Tongue Cancer Treated With Surgery Alone
Source: Kaohsiung J Med Sci. 2025 Sep 1;42(2):e70102. doi: 10.1002/kjm2.70102 (PMC12884757; doi:10.1002/kjm2.70102)

Figure S1. Distribution of tumor’s depth of invasion in our cohort.


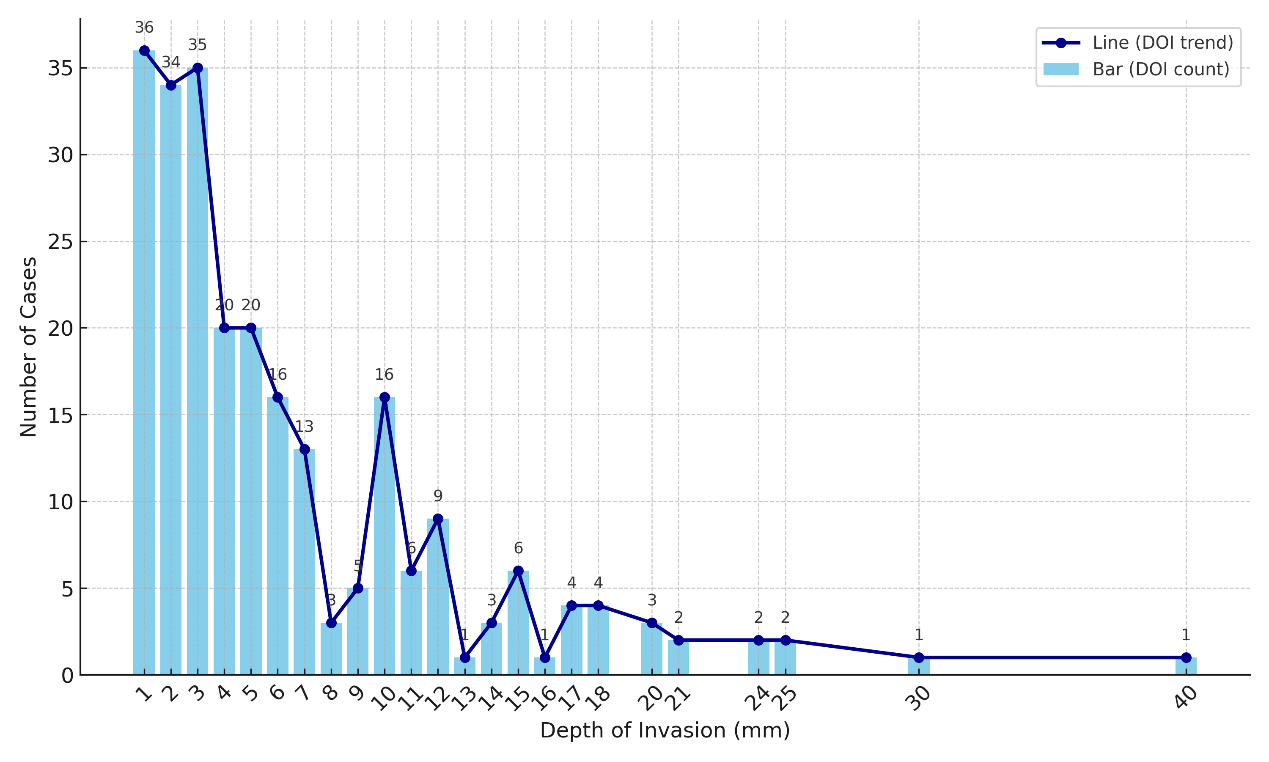


Figure S2. Regional recurrence rate based on cumulative depth of invasion in subgroup patients with cN0 disease who underwent a watchful waiting strategy for neck management.


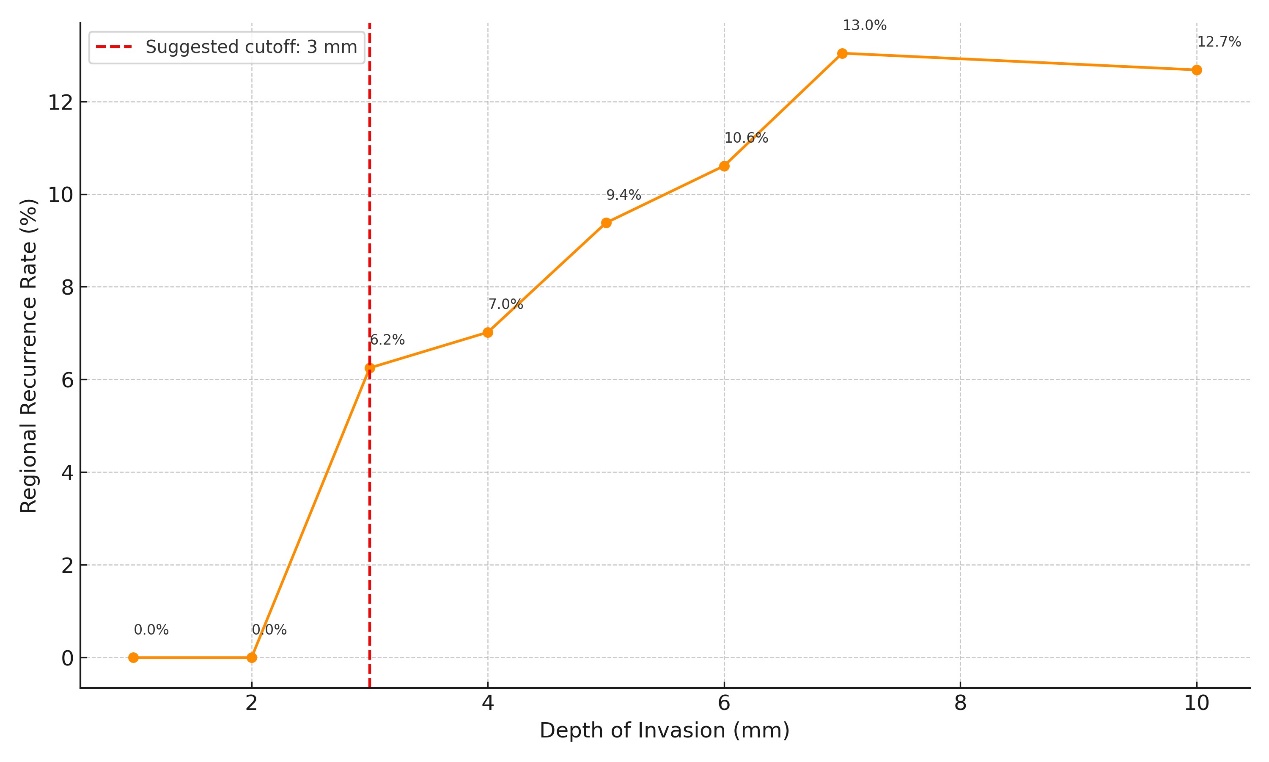

Supplement: Supplementary file 1 — Figure S1: Distribution of tumor's depth of invasion in our cohort. Figure S2:. Regional recurrence rate based on cumulative depth of invasion in subgroup patients with cN0 disease who underwent a watchful waiting strategy for neck management. [file KJM2-42-e70102-s001.docx]
